# Th1-Biased Hepatitis C Virus-Specific Follicular T Helper-Like Cells Effectively Support B Cells After Antiviral Therapy

**DOI:** 10.3389/fimmu.2021.742061

**Published:** 2021-09-30

**Authors:** Katharina Zoldan, Sabine Ehrlich, Saskia Killmer, Katharina Wild, Maike Smits, Marissa Russ, Anna-Maria Globig, Maike Hofmann, Robert Thimme, Tobias Boettler

**Affiliations:** ^1^ Department of Medicine II, University Hospital Freiburg, Faculty of Medicine, University of Freiburg, Freiburg, Germany; ^2^ Faculty of Chemistry and Pharmacy, University of Freiburg, Freiburg, Germany; ^3^ Faculty of Biology, University of Freiburg, Freiburg, Germany

**Keywords:** immunology and infectious diseases, adaptive immunity, cellular immune response, MHC class II, T cell immunity

## Abstract

Circulating Th1-biased follicular T helper (cTfh1) cells have been associated with antibody responses to viral infection and after vaccination but their B cell helper functionality is less understood. After viral elimination, Tfh1 cells are the dominant subset within circulating Hepatitis C Virus (HCV)-specific CD4 T cells, but their functional capacity is currently unknown. To address this important point, we established a clone-based system to evaluate CD4 T cell functionality *in vitro* to overcome experimental limitations associated with their low frequencies. Specifically, we analyzed the transcription factor expression, cytokine secretion and B cell help in co-culture assays of HCV- (n = 18) and influenza-specific CD4 T cell clones (n = 5) in comparison to Tfh (n = 26) and Th1 clones (n = 15) with unknown antigen-specificity derived from healthy donors (n = 4) or direct-acting antiviral (DAA)-treated patients (n = 5). The transcription factor expression and cytokine secretion patterns of HCV-specific CD4 T cell clones indicated a Tfh1 phenotype, with expression of T-bet and Bcl6 and production of IFN-γ and IL-21. Their B helper capacity was superior compared to influenza-specific or Tfh and Th1 clones. Moreover, since Tfh cells are enriched in the IFN-rich milieu of the HCV-infected liver, we investigated the impact of IFN exposure on Tfh phenotype and function. Type I IFN exposure was able to introduce similar phenotypic and functional characteristics in the Tfh cell population within PBMCs or Tfh clones *in vitro* in line with our finding that Tfh cells are elevated in HCV-infected patients shortly after initiation of IFN-α therapy. Collectively, we were able to functionally characterize HCV-specific CD4 T cells *in vitro* and not only confirmed a Tfh1 phenotype but observed superior Tfh functionality despite their Th1 bias. Furthermore, our results suggest that chronic type I IFN exposure supports the enrichment of highly functional HCV-specific Tfh-like cells during HCV infection. Thus, HCV-specific Tfh-like cells after DAA therapy may be a promising target for future vaccination design aiming to introduce a neutralizing antibody response.

**Graphical Abstract:**
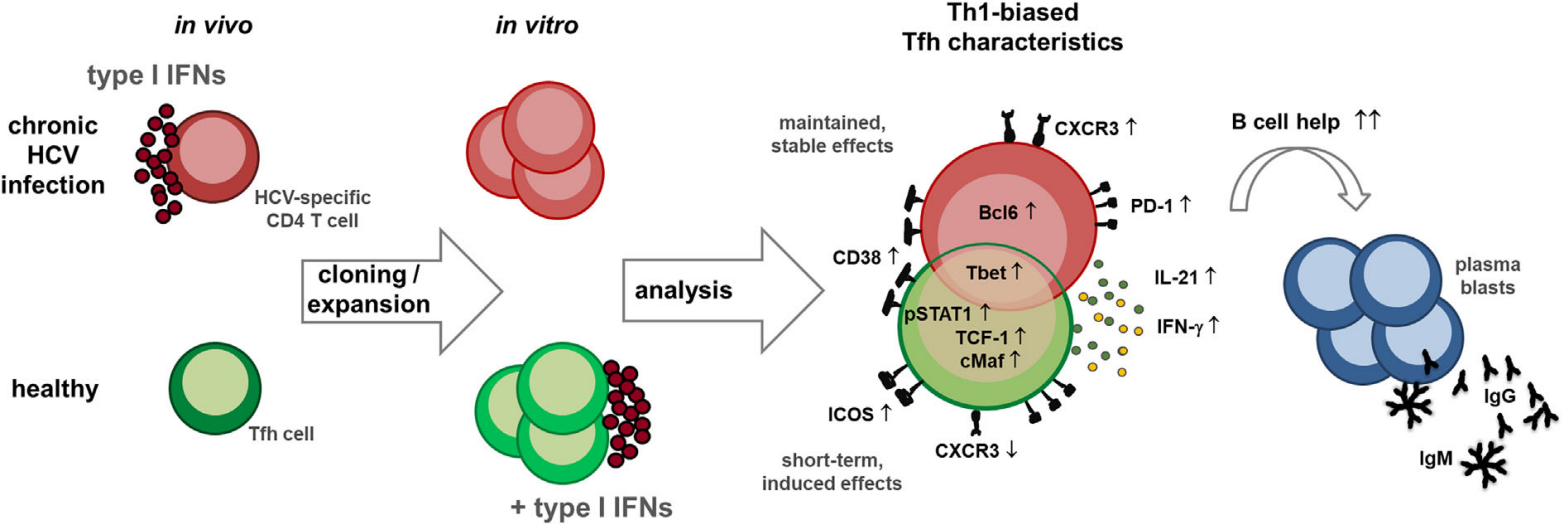


## Introduction

CD4 T cells are the central regulators of the virus-specific cellular and humoral immune response [reviewed in (1–3)] as their deletion leads to viral persistence in animal models (4, 5) whereas clearance of HCV infection is associated with a sustained multi-specific CD4 T cell response (6, 7). In mice, persistent viral infection drives the formation of a virus-specific helper T cells type 1 (Th1)-pool and a phenotypically and functionally distinct follicular T helper (Tfh) cell-pool (8, 9). While Th1 cells provide help to sustain antiviral CD8 T cell responses, Tfh cells are specialized in B cell support to mount a strong antibody response that is critical for viral control. In humans, less is known about the presence and functionality of Tfh cells during viral infections, especially during HCV infection, as the peripheral HCV-specific CD4 T cell population rapidly collapses with progression to chronicity (7). However, CD4 T cells with a Tfh phenotype accumulate in the chronically infected liver tissue (10, 11).

Human Tfh cells that express the surface receptors CXCR5 and PD-1 but lack the expression of CXCR3 are known as circulating counterparts of germinal center Tfh cells (cTfh), are able to provide help to memory B cells *in vitro* and are associated with neutralizing HIV antibody responses (12), whereas CXCR3+ Th1-biased cTfh (cTfh1) cells may either fail to support memory B cells *in vitro* (12, 13) but have also shown to correlate with antibody responses to HIV (14, 15), HCV (16–18), other viral infections (19) and after vaccination (20–23) in humans and animal models. Most recently, the early activation of CXCR3+ but not CXCR3- Tfh cells has been associated with more effective antibody responses and resolution of HCV infection (24). In addition, it has been shown that they display an equal capacity to support B cells *in vitro* compared to CXCR3- Tfh cells (16, 25). Collectively, the role of cTfh1 cells during viral infections in humans is incompletely understood. Furthermore, as virus-specific Th1 and Tfh cells appear to form distinct phenotypes in LCMV-infected mice, the processes that mediate the formation of the Tfh1 phenotype during chronic viral infection in humans remain elusive. In a previous study conducted by us, *ex vivo* flow cytometric and gene expression analysis revealed a mixed Tfh and Th1 phenotype to be the dominant subset within circulating HCV-specific CD4 T cells after viral elimination by direct acting antiviral (DAA) therapy (26). Based on this data set we aimed for the functional validation in our current study. The characterization of their Tfh functionality and the identification of the mechanisms that drive their formation may help to establish them as targets for future vaccination strategies that aim to introduce neutralizing antibodies against HCV.

## Materials and Methods

### Study Subjects

This study was approved by the ethics committee of the Albert Ludwig University Freiburg (344/13, 507/19 and 227/15). All individuals gave written informed consent prior to donating blood for the biobank. 5 cHCV-infected, DAA-treated patients, 7 cHCV-infected patients undergoing PEG-IFN-α treatment and 13 healthy donors (HD) were enrolled in this study. For detailed information on sampling time points and donor characteristics see **Table 1**. Relevant individuals were HLA-typed by next generation sequencing using commercially available primers (GenDx, Utrecht, The Netherlands). Samples were run on a MiSeq system. NGSengine^®^ Software (GenDx) was used for data analysis.

**Table 1:** Study cohort and PBMC donors of cloned cells.

Donor number	age/years	Cohort	Sex	Genotype	Therapy	SVR	sampling time points	Analyzed cells/clones	relevant HLA type
1	32	HD	f	–	–	–	–	B cells, PBMCs 2 Tfh clones	–
2	38	HD	m	–	–	–	–	clones: total 12 (5 Flu-specific, 6 Tfh, 1 Th1)	DRB1*0101
3	28	HD	f	–	–	–	–	clones: total 7 (5 Tfh, 2 Th1)	–
4	35	HD	f	–	–	–	–	clones: total 4 (2 Tfh, 2 Th1)	–
5	53	HD	m	–	–	–	–	PBMCs	–
6	38	HD	m	–	–	–	–	PBMCs	–
7	28	HD	f	–	–	–	–	PBMCs	–
8	27	HD	f	–	–	–	–	PBMCs	–
9	23	HD	f	–	–	–	–	PBMCs	–
10	27	HD	f	–	–	–	–	PBMCs	–
11	24	HD	f	–	–	–	–	PBMCs	–
12	22	HD	f	–	–	–	–	PBMCs	–
13	30	HD	m	–	–		–	PBMCs	–
14	52	HCV DAA	f	1a	Glecaprevir/Pibrentasvir	yes	5 month after EOT	clones: total 1 (1 HCV-specific)	DRB1*0101
15	52	HCV DAA	f	1b	Glecaprevir/Pibrentasvir	yes	4 month after EOT	clones: total 5 (3 Tfh, 2 Th1)	DRB1*15:01
16	62	HCV DAA	m	1a	Elbasvir/Grazoprevir	yes	11 month after EOT	clones: total 10 (4 HCV-specific, 3 Tfh, 3 Th1)	DRB1*15:01
17	45	HCV DAA	f	1b	Glecaprevir/Pibrentasvir	yes	7 month after EOT	clones: total 4 (2 Tfh, 2 Th1)	–
18	40	HCV DAA	f	1b	Elbasvir/Grazoprevir	–	week 4 of therapy	clones: total 19 (13 HCV-specific, 3 Tfh, 3 Th1)	DRB1*0101
19	36	HCV PEG	f	6	Peg-IFN-α	yes	BL; W12; W40	PBMCs	–
20	66	HCV PEG	m	1b	Peg-IFN-α, Ribavirin & Sofosbuvir	yes	BL; W12; FU24	PBMCs	–
21	39	HCV PEG	m	3a	Peg-IFN-α & Ribavirin	yes	BL; W11; W17; longterm FU	PBMCs	–
22	43	HCV PEG	m	1b	Peg-IFN-α, Ribavirin & Sofosbuvir	yes	BL; W12	PBMCs	–
23	35	HCV PEG	m	1b	Peg-IFN-α, Telaprevir, Ribavirin	yes	BL; W12	PBMCs	–
24	40	HCV PEG	m	1b	Peg-IFN-α, Telaprevir, Ribavirin	yes	BL; W4, W45, FU12	PBMCs	–
25	22	HCV PEG	m	3b	Peg-IFN-α & Ribavirin	yes	BL; W4; W24, FU24	PBMCs	–

DAA, direct acting antivirals; PEG, pegylated IFN-α; BL, baseline; w, week; EOT, end of treatment; FU, follow up; HD, healthy donor; SVR, sustained virological response.

### Study Design

To provide sufficient cell numbers for functional assays, peripheral HCV-specific CD4 T cells were cloned and analyzed *in vitro*. Since primary T cells are known to quickly alter their characteristics during *in vitro* cultivation, we aimed to analyze the stabile characteristics that are maintained *in vitro*. For this purpose the HCV clones were compared to reference CD4 T cell clone types. The reference system consisted of CD4 T cell clone types of different immunological backgrounds (non-persistent vs. persistent viral infection) and of Tfh and Th1 background since these lineages are most relevant during the antiviral response.

cTfh cells derive from germinal center (GC) Tfh cells, what has been demonstrated by their clonal relation (27–29) and for establishing the Tfh clones, we used cTfh cells of the phenotype that transcriptionally and also functionally most closely resembles GC Tfh cells (12).

We established HCV clones (background of persistent infection) from three patients during or after DAA therapy. Flu clones derived from one HD provided the background of a non-persistent viral infection. Tfh and Th1 clones derived from five HCV-infected patients and four HD and had unknown antigen-specificity. Details on the *ex vivo* phenotypes of the cloned single cells are summarized in **Tables 2** and **3**. **Supplementary Table 1** summarizes all CD4 T cell clones included in the study.

**Table 2:** Sorting phenotype of CD4 T cell clones.

Clone type	*sorting* phenotype
HCV	non-naive, CD4+, Tet+ (phenotypic characterization: (26)
Flu	CD45RA-, CD4+, Tet+: all CXCR3+, see **Table 3**: index sort
Tfh	sort gate: CD45RA-, CXCR5+, PD-1+, CXCR3-
Th1	sort gate: CD45RA-, CXCR5-, CCR6-, CXCR3+

non-naive: CD45RA-CCR7+ and CD45RA-CCR7- and CD45RA+, CCR7-, Tet, Tetramer.

**Table 3:** Index-sorting phenotype of Flu clones.

Clone	CXCR5	CXCR3	PD-1
2FluA9	+	+	–
2FluH2	+	+	–
2FluA7	+	+	–
2FluC1	–	+	+
2FluE2	–	+	+

To ensure direct comparability of all clones all analyses were conducted within the same experimental run. All clones received the same pre-treatment prior to analysis (see methods section for details) to adapt the cultures to an equal level of activation, metabolic state and vitality. The number of restimulations (see culture methods for details) per clone was between 6 and 13 prior to analysis.

To validate our methodological approach, we compared the phenotypic and functional characteristics of Tfh and Th1 clones as the lineage differences are well described (8, 12, 30). Relevant surface markers and transcription factors that are associated with CD4 T cell lineage or T cell activation were included. Since human Tfh cells rapidly loose expression of chemokine receptor CXCR5 *in vitro* (31–33) and CXCR5 is negatively regulated by IL-2 on Tfh cells (34), this lineage-defining surface marker was not included in our analysis. **Supplementary Figure 5** shows the direct comparison of parameters that best discriminate Tfh and Th1 clones in our *in vitro* approach.

The *in vitro* maintenance of the main lineage-specific phenotype and functionality of Tfh and Th1 clones demonstrates the validity of our clone-based approach. Indeed, lineage commitment of Tfh and Th1 cells has been described during viral re-challenge in mice (8). Thus, our *in vitro* system allowed the evaluation of phenotype and functionality of HCV clones by analysis of basic stabile characteristics that are maintained *in vitro* and direct comparison between the CD4 T cell clone types. Direct comparability was ensured by the synchronization of the cell culture conditions and treatment.

### Cytokines and Stimulants

Used cytokines and (co)stimulants are listed in **Supplementary Table 2**.

### Generation of CD4 T Cell Clones

Isolation of PBMCs, preparation of feeder cells and generation of CD4 T cell clones are described in detail in the **Supplementary Methods** section.

### CD4 T Cell Clones

**Figure 1:**
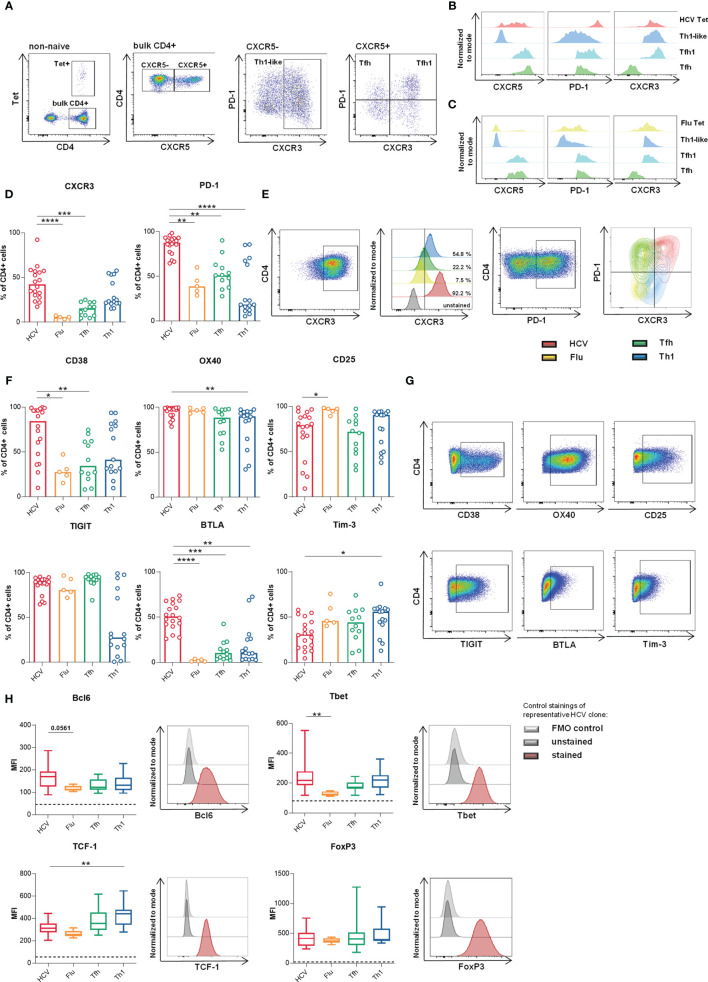
HCV clones present phenotypic and transcriptional Th1-biased Tfh-like characteristics. **(A–C)** Representative dotplots and histograms of *ex vivo* analyzed HCV and Flu-specific CD4 T cells in the peripheral blood (donor 2 and 17 respectively) after magnetic bead based tetramer enrichment as described previously (26, 35). **(D–G)** Surface marker expression on the clone types was analyzed by flow cytometry (n = 18 HCV, 5 Flu, 12 Tfh, 15 Th1 clones). **(D)** CXCR3 and PD-1 expression. **(E)** Stainings of data in **(D)** of representative clones as pseudocolor plots, histograms and overlaid contour plots, respectively. **(F)** Expression of activation and inhibitory markers among the clone types. **(G)** Representative stainings from data shown in **(F)**. **(D, F)** Bars represent the median. Each symbol represents one CD4 T cell clone of the indicated type. **(H)** Median expression (MFI) of relevant CD4 T cell lineage-associated transcription factors was analyzed by flow cytometry (n = 18 HCV, 5 Flu, 14 Tfh, 13 Th1 clones) and is shown as box plot with minimum to maximum whiskers. Dashed lines represent the MFI of unstained controls. A representative staining with unstained and fluorescence minus one (FMO) control of one HCV clone is shown on the right side of each box plot. **(D–H)** Kruskal-Wallis test with Dunn’s test for multiple comparisons was applied to compare differences between all groups to HCV clones as control. *p < 0.05; **p < 0.01; ***p < 0.001; ****p < 0.0001.

**Figure 2:**
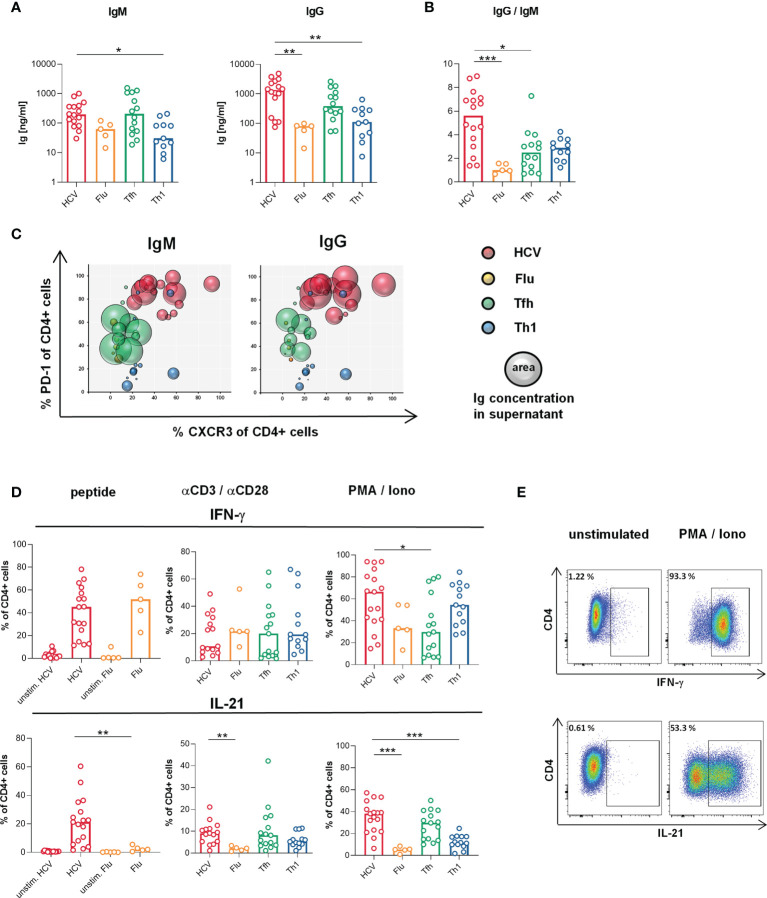
Superior B cell helper capacity despite Th1-biased cytokine production of HCV clones. **(A, B)** B cell helper capacity was analyzed by coculture of the CD4 T cell clones (n = 16 HCV, 5 Flu, 14 Tfh, 11 Th1 clones) with naive allogeneic B cells for 7 d **(A)** Concentrations of IgM and IgG in cell-free coculture supernatants were determined by ELISA. **(B)** Class switch induced by the CD4 T cell clone types as ratio of IgG and IgM concentration in culture supernatants. For coculture system validation plasmablast formation was correlated with produced Ig **(Supplementary Figure 7A)**. B cell positive and negative controls and autologous in-run controls are shown in **Supplementary Figures 7C, D**, respectively. **(C)** The correlation of CXCR3 and PD-1 surface expression (shown in **Figures 1D**) with the individual B cell helper capacity is visualized in bubble plots (n = 16 HCV, 5 Flu, 11 Tfh, 11 Th1 clones). **(D)** Production of IFN-γ and IL-21 by the CD4 T cell clone types was determined by flow cytometry after stimulation with their respective peptide (n = 17 HCV, 15 unstim. HCV, 5 Flu, 5 unstim. Flu clones), αCD3/αCD28 (n = 15 HCV, 5 Flu, 15 Tfh, 13 Th1 clones) or PMA/Iono (n = 17 HCV, 5 Flu, 15 Tfh, 13 Th1 clones). **(E)** Representative pseudocolor plots of cytokine stainings after PMA/Iono stimulation. **(A, B, D)** Bars indicate the median. Each symbol represents one CD4 T cell clone of the indicated type. Kruskal-Wallis test with Dunn’s test for multiple comparisons was applied to compare differences between all groups to HCV clones as control. Mann-Whitney rank sum test was used to compare two groups. *p < 0.05; **p < 0.01; ***p < 0.001.

**Figure 3:**
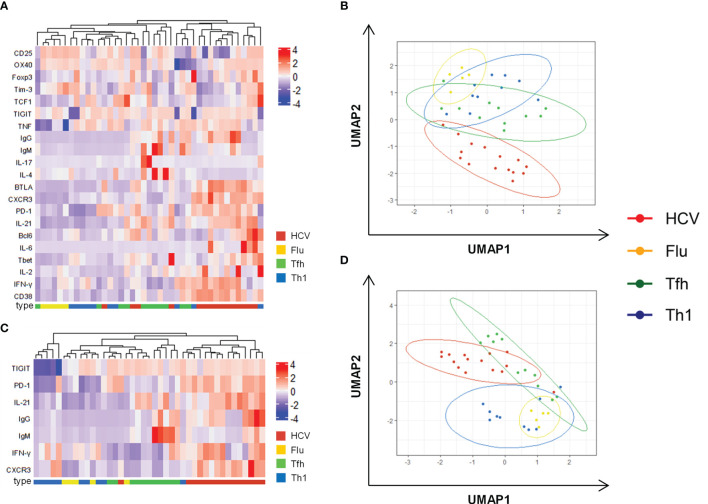
Combined analysis confirms unique Th1-biased Tfh characteristics of HCV clones. **(A)** Heatmap with unbiased clustering analysis (rows and columns) of the clone types including all analyzed parameters. **(B)** Corresponding UMAP analysis of data in **(A)**. **(C)** Heatmap with unbiased clustering analysis (rows and columns) of the clone types including parameters that are able to discriminate between Tfh and Th1 clones **(Supplementary Figure 5)**. **(D)** Corresponding UMAP analysis of data in **(C)**. **(A–D)** Cytokine production after stimulation with PMA/Iono was used for these analyses. Only clones with a complete analysis of all parameters were included (n = 15 HCV, 5 Flu, 11 Tfh, 10 Th1 clones). **(B, D)** Each symbol represents one CD4 T cell clone of the indicated type.

Newly generated and previously established CD4 T cell clones were used [(34) and, **Supplementary Table 1**]. The donor (**Table 1**) from which each clone was generated, is recorded in the first number of the clone’s name. The generation of CD4 T cell clones is described in the **Supplementary Methods** part. The CD4 T cell clone types were cultured in AIM-V cell culture medium supplemented with 5 U/ml IL-2. Every 14 days the cultures were restimulated with 2 µg/ml PHA, 5 U/ml IL-2 and 6x10^6^ feeder cells in T75 flasks. IL-2 was added to the cultures every 3 to 4 days. Medium was added on demand. Prior to experiments all clone cultures were adjusted to an equal level of activation: The cultures were supplemented with an equal volume of fresh medium and IL-2 on day 3 and day 6 after restimulation. For analyses the cells were used on day 6 before addition of medium and IL-2 (flow cytometry) and day 7 (coculture experiments) after restimulation. To ensure comparability of the results shown in **Figures 1**–**3** only clone cultures from the same batch (i.e. originating from the same restimulation mixture) were analyzed within the same experimental run.

### IFN Stimulation of PBMCs

PBMCs were isolated from EDTA anti-coagulated blood and used immediately. 0.5x10^6^ cells were plated in each well and incubated overnight at 5 % CO_2_ and 37°C. The following day, the different IFNs were added at a concentration of 0.5 pmol/ml. The cells were then incubated at 5 % CO_2_ and 37°C for 3 d and subsequently used for flow cytometric staining.

### IFN Stimulation of Tfh Clones

Tfh clone cells were used for experiments within the second week after their last restimulation (see *in vitro* culture). For the analysis of transcription factor expression 0.3x10^6^ clone cells were seeded and incubated at 5% CO_2_ and 37°C for 3 d in the presence of αCD3/αCD28 (T cell activator) and 0.5 pmol/ml of the different IFNs. For the analysis of cytokine production 0.5x10^6^ cells were seeded and incubated at 5% CO_2_ and 37°C for 5 h in the presence of 0.5 pmol/ml of the different IFNs, αCD3/αCD28 (T cell activator) and 0,325 µl/ml GolgiStop™ and 0,5 µl/ml GolgiPlug™ (BD Biosciences). Subsequently, the cells were used for flow cytometric staining.

### Flow Cytometric Analysis

Clone cells or PBMCs were surface stained or fixed and permeabilized to stain intracellular or intranuclear targets with specific tetramers or antibodies (**Supplementary Tables 3** and **4**). **Supplementary Methods** contain detailed procedures.

### T Cell-B Cell Coculture

After isolation of PBMCs naïve B cells were isolated by negative magnetic selection using the naïve B cell Isolation Kit (Stemcell Technologies, France). 30,000 B cells were seeded 1:1 with CD4 T cell clone cells in 96-well V-bottom plates. Clone cells were activated by 10 µl/ml αCD3/αCD28 (T cell activator, Stemcell Technologies). The volume was adjusted to 200 µl with coculture medium (IMDM medium, 10 % FCS). B cells alone or stimulated with 25 ng/ml IL-21 and 100 ng/ml MEGACD40L served as control. The cultures were incubated for 7 d. After incubation culture supernatants were frozen and cells were stained with surface antibodies.

### Enzyme-Linked Immunosorbent Assay

IgG and IgM in coculture supernatants were quantified by ELISA as described previously (36). Antibodies were obtained from Jackson ImmunoResearch, Dianova, Germany. In brief, Nunc MaxiSorp Plates (VWR, Germany) were coated with anti-human IgG (heavy chain + light chain) antibody. Human IgG and IgM (whole molecule) in defined concentrations served as standard curve. Peroxidase-conjugated anti-human Fc5µ or Fcgamma F(ab’)2 fragments were applied were applied for detection. Absorption at 450 and 570 nm was measured with a TECAN Spark Multiwell Plate Reader (TECAN GmbH, Crailsheim, Germany).

### Antiviral Activity of the Used IFN Reagents

For analysis of the antiviral activity of the IFN reagents the HCV replicon system was used. Details are explained in the **Supplementary Methods** part.

### Statistical Analysis

For graphical visualization and statistical analyses GraphPad Prism 8 software (GraphPad Software, San Diego, USA) was used. Unpaired data were analyzed using non-parametric testing with Kruskal-Wallis test and Dunn’s test for multiple comparisons. For comparison of two groups Mann-Whitney rank sum test was used. Paired data were analyzed using non-parametric testing with Friedman test and Dunn’s test for multiple comparisons. For comparison of two groups Wilcoxon matched-pairs signed rank test was used. Levels of significance are indicated as follows: * p < 0.05, ** p < 0.01, *** p < 0.001, **** p < 0.0001. Bubble plots were generated using Microsoft Excel (Microsoft Corporation, Redmond, USA). For dimension reduction uniform manifold approximation and projection (UMAP) technique was applied. For UMAP analysis and graphical visualization R Studio (https://www.r-project.org) was used. The UMAP plot was generated using the UWOT package with scaled values and n_neighbors = 10. Ellipses were generated with the 0.95 confidence interval. The ComplexHeatmap package was used to generate heatmaps (37).

## Results

### HCV Clones Present Phenotypic and Transcriptional Th1-Biased Tfh-Like Characteristics

*Ex vivo* HCV-specific CD4 T cells display a memory-like phenotype with overlapping Tfh/Th1 signatures after DAA therapy (26). The expression levels of the main surface markers characterizing peripheral Tfh and Th1 cells (i.e. CXCR5, PD-1 and CXCR3) are shown on HCV-specific CD4 T cells in comparison to influenza- (Flu-) specific CD4 T cells *ex vivo* in a representative HCV-infected, DAA-cured or healthy donor, respectively (**Figures 1A–C**). To facilitate the functional analysis of the scarce HCV-specific CD4 T cell population (**Figure 1A**), we generated HCV-specific CD4 T cell clones from these patients. In a first step, we characterized their phenotype and transcription factor expression since primary T cells are known to quickly alter their phenotype during *in vitro* cultivation. To allow evaluation of HCV-specific CD4 T cell clones with this approach, we used a reference system with three additional CD4 T cell clone types for comparison. Virus-specific CD4 T cell clones derived from circulating HCV- (red) or influenza (Flu)-specific (yellow) CD4 T cells were generated using MHC class II tetramers. CD4 T cell lineage-specific clones were derived from circulating Tfh (green) or Th1 (blue) cells with unknown antigen-specificity (see methods section for details on the viral epitopes, cloning procedures and culture conditions). Direct comparability of all clone cultures was ensured by the synchronization of the cell culture conditions and treatment. The *in vitro* maintenance of the main lineage-specific phenotype and functionality of Tfh and Th1 clones demonstrated the validity of this clone-based approach (**Supplementary Figure 5**). Since human Tfh cells rapidly loose expression of chemokine receptor CXCR5 *in vitro* (31–33) and CXCR5 is negatively regulated by IL-2 on Tfh cells (34), this lineage-defining surface marker was not included in our analysis (see study design in the methods section for more details).

Besides their *ex vivo* CXCR5 expression or virus-specificity, the single cells were mainly distinguished by their *ex vivo* CXCR3 and PD-1 expression (**Tables 2** and **3**) characterizing the Th1 and Tfh phenotype, respectively, prior to cloning. These basic differences were maintained comparing Tfh and Th1 clones (**Supplementary Figure 5** and **Figure 1D**). Furthermore, HCV clones showed high expression of both markers as expected from the *ex vivo* analysis of HCV-specific CD4 T cells after DAA treatment (26) demonstrating the maintenance of high levels of CXCR3 and PD-1 expression and a Th1-biased Tfh phenotype. However, Flu clones, which were all CXCR3+ at cloning (**Table 3**) showed reduced CXCR3 and a moderate PD-1 expression compared to Tfh and Th1 clones (**Figures 1D, E**).

To better characterize our *in vitro* system, we analyzed the phenotype of all clone types more deeply, including surface markers and transcription factors that characterize CD4 T cell lineages or are associated with activation and exhaustion of T cells during chronic viral infection. High CXCR3, CD38 and OX40 expression indicated a high level of activation but also inhibitory surface markers like PD-1, TIGIT and BTLA were highly expressed by HCV clones (**Figures 1F, G**). Their strong TIGIT expression level that was comparable to Tfh clones further indicated Tfh features (12). OX40 and CD25 were highly expressed among all clone types indicating that their upregulation by the IL-2-rich culture conditions (34) outbalanced possible cell type-specific differences.

On the transcriptional level the concurrent expression of B cell lymphoma 6 (Bcl6) and T-box transcription factor (Tbet) within HCV clones (**Figure 1H**) supported a Th1-biased Tfh-like profile.

T cell factor 1 (TCF-1), which is a key regulator of Tfh differentiation upstream of Bcl6 (38, 39) but also drives the maintenance of T cell effector functions during chronic viral infections (40–42), was more strongly expressed in Tfh and Th1 clones in comparison to HCV and Flu clones.

Furthermore, the addition of IL-2 in the culture conditions did not appear to differentially regulate FoxP3 expression, which was similarly expressed in all clone types, excluding an individual Treg bias of one clone type induced by the culture conditions [reviewed in (43)]. Overall, Flu clones showed low expression of the analyzed transcription factors (**Figure 1H**).

Collectively, clone type-specific differences were detected for most of the analyzed markers. Based on surface marker and transcription factor expression, we observed Th1-biased Tfh phenotype and transcriptional activity among the HCV clones, whereas Flu clones rather showed a Tfh-like phenotype with lower expression of lineage-defining transcription factors.

### HCV Clones Possess Superior B Cell Helper Capacity Despite Th1-Biased Cytokine Production

Next, we addressed whether the Th1-biased Tfh phenotype of the HCV clones is reflected in their functionality. As the most decisive feature of Tfh functionality, B cell helper capacity was analyzed *in vitro* by coculture of the CD4 T cell clones with naïve allogeneic B cells. HCV clones showed superior ability to support B cells during 7 d incubation resulting in higher production of IgM and IgG (**Figure 2A**) and a better class switch to IgG (**Figure 2B**), when compared to Flu or Th1 clones. To correlate the B cell helper capacity with the Tfh/Th1 phenotype of the clone types on an individual level, we used bubble plots (**Figure 2C**). The expression of PD-1 and CXCR3 (in **Figure 1D**) were chosen for this analysis as these surface markers characterize a predominant Tfh versus Th1phenotype in our *in vitro* system. Tfh clones with high B cell helper capacity expressed lower levels of CXCR3 and more PD-1, whereas Th1 clones predominantly expressed more CXCR3 and low PD-1 and showed lower B cell helper capacity, as expected. Interestingly, Flu clones showed low B cell help despite an overlapping CXCR3 and PD-1 expression with Tfh clones that showed higher B cell help. Strikingly, HCV clones showed superior B cell helper capacity despite expression of high levels of CXCR3 and PD-1 indicating that their phenotypic Th1 bias did not affect their B cell helper functionality.

In addition to their B cell helper capacities, we analyzed the cytokine production of the different T cell clones to further evaluate their lineage commitment and to determine the possible impact of the culture conditions. All clone types secreted IFN-γ, IL-21 and TNF (**Figures 2D, E** and **Supplementary Figure 6**) with the most striking differences in IL-21 and IFN-γ production (**Figure 2D**). In response to stimulation with their specific peptide or PMA/Ionomycin, HCV clones co-produced high levels of IL-21 and IFN-γ, which were comparable to those of Tfh and Th1 clones, respectively, confirming a Tfh/Th1 overlap on the functional level. Other cytokines such as IL-2, IL-4, IL-6 and IL-17 were produced at lower levels (**Supplementary Figure 6**).

Collectively, the B cell helper capacity of HCV clones was superior compared to the other clone types despite their Th1-biased Tfh phenotype and cytokine production.

### Combined Analysis Confirms Major Tfh Characteristics With Th1-Bias of HCV Clones

To summarize the analysis of the CD4 T cell clone types we used heatmaps with unbiased clustering and dimension reduction by uniform manifold approximation and projection (UMAP) visualization. Analysis of all phenotypic and functional parameters resulted in a separation of HCV and Flu clones with Tfh and Th1 clones as intermediate phenotypes (**Figures 3A, B**). The clustering of the parameters and the clone types is further reflected by their significant correlation (**Supplementary Figure 9**). To better characterize the CD4 T cell subset of the virus-specific clones, markers that separated Tfh and Th1 clones were included in a second analysis (**Figures 3C, D**). Again, HCV clones located to different areas of the map compared to Flu clones and showed some overlap with Tfh but not Th1 clones. However, their localization between Tfh and Th1 clones indicated an intermediate phenotype while the localization of the Flu clones rather pointed towards Th1 characteristics of the Flu clones (**Figure 3D**).

Collectively, HCV clones showed functional and phenotypic characteristics of both Tfh and Th1 lineages. The Th1 bias within their surface marker and transcription factor expression as well as their cytokine production did not seem to negatively influence their Tfh functionality *in vitro*. However, the underlying mechanisms that shape and maintain this Tfh-like phenotype during chronic HCV infection remain poorly understood.

### Type I IFNs Support Maintenance, Activation and Functionality of Tfh Cells

HCV clones showed unique characteristics that were maintained during *in vitro* culture. In the context of CD8 T cell exhaustion, it is well established that the immunologic environment of chronic viral infection, that involves chronic exposure to antigen stimulation and IFN signaling, can stably and irreversibly imprint phenotypic and functional characteristics (44, 45) and we hypothesized that similar effects could be elicited in CD4 T cells. Moreover, Tfh cells are enriched in the HCV-infected liver tissue (10, 11) which is known to express high levels of IFN-induced genes (46). Hence, we investigated the influence of IFN exposure as possible mediator for stabilization of Tfh-like features.

**Figure 4:**
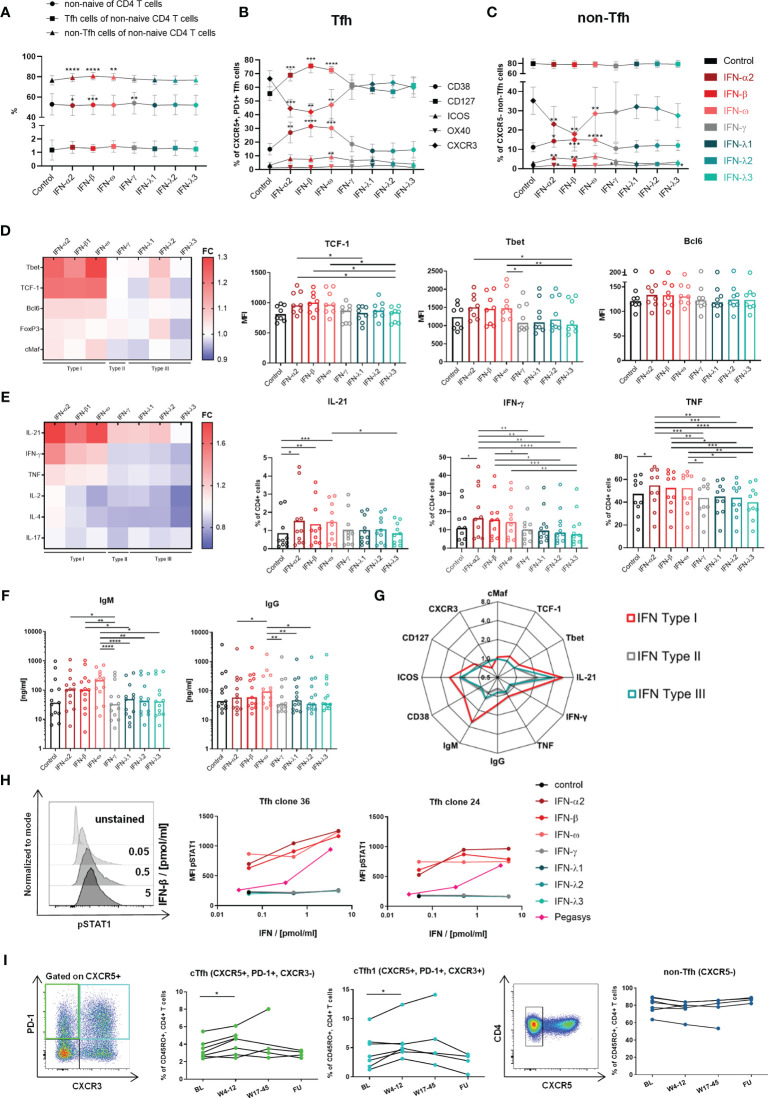
Type I IFNs introduce a Th1-bias and enhance activation and functionality of Tfh cells. **(A–C)** Changes on non-naive CD4 T cells, non-Tfh- and Tfh cells were analyzed within HD PBMCs (n = 10) after incubation in the indicated conditions for 3 d **(A)** Median and 95% confidence interval (CI) for analyzed cell populations. **(B)** Expression of activation and costimulatory markers on Tfh **(B)** and non-Tfh cells **(C)** as median with CI. The gating strategy is shown in **Supplementary Figure 2**. **(A–C)** Friedman test with Dunn’s test for multiple comparisons were applied to compare all conditions to control. **(D)** Transcription factor expression within Tfh clones (n = 8) was analyzed after 3 d incubation in the indicated conditions in the presence of αCD3/αCD28 stimulation. For representative stainings see **Figure 1**. **(E)** Cytokine production of Tfh clones (n = 10) was analyzed after 5 h incubation in the indicated conditions and stimulation with αCD3/αCD28. For representative stainings see **Figure 2** and **Supplementary Figure 6**. Heatmaps show the fold change (FC: mean expression normalized to control). **(D, E)** Bars show the median. One symbol represents one clone. **(F)** B cell helper capacity was analyzed by 7 d coculture of Tfh clones with autologous naive B cells in the indicated conditions. Ig concentrations in coculture supernatants were determined by ELISA. Bars show the median. One symbol represents the mean of technical duplicates. Negative and positive controls for B cells are shown in **Supplementary Figures 7E, F**. **(G)** Summarized changes within Tfh cells and Tfh clones introduced by type I IFN exposure. The mean FC against the control condition is shown for Type I, II and III IFN influence. **(D–F)** Friedman test with Dunn’s test for multiple comparisons were applied to compare one condition to every other condition. **(H)** Tfh clones were incubated with the respective IFN in the indicated concentrations for 20 min. Phosphorylation of STAT1 (pSTAT1) was determined by BD PhosFlow™. Mean fluorescence intensity (MFI) of pSTAT1 with respresentative histograms. Gated on CD4+ cells. **(I)** PBMCs of cHCV patients under therapy with pegylated IFN-α were analyzed for changes in cTfh, cTfh1 and non-Tfh cell frequencies. One symbol represents one patient. Representative pseudocolor plots on the left. All time points were compared to BL using the Wilcoxon matched-paires signed rank test with Bonferroni correction (p < 0.016). BL, baseline; W, week; FU, follow up, *p < 0.05; **p < 0.01; ***p < 0.001; ****p < 0.0001.

The influence of type I IFNs was analyzed using an *in vitro* approach in comparison to type II and III IFNs, which are other components of the IFN response. The functionality of the used IFN reagents was confirmed in advance by assessment of the antiviral activity using an established assay with HCV replicon-transfected Huh7 cells (47, 48) (**Supplementary Figure 3**). To mimic the influence of an altered peripheral cytokine milieu, isolated HD PBMCs were incubated in the presence of different IFNs for 3 d. While the frequencies of non-naïve CD4 T cells, non-Tfh and Tfh cells showed only minor alterations (**Figure 4A**), activation markers like CD38 and ICOS as well as the costimulatory IL-7 receptor α (CD127) were significantly upregulated and CXCR3 was downregulated on Tfh cells after type I but not type II or III IFN exposure (**Figure 4B**). We observed a similar pattern of regulation on non-Tfh cells, although to a lesser extent (**Figure 4C**). Although the downregulation of CXCR3 expression was surprising given the high levels of CXCR3 expression on HCV-specific CD4 T cells *ex vivo* (26) and after cloning, these experiments suggest that type I IFNs in particular may be involved in shaping the phenotype of CD4 T cells. To evaluate the effects of IFNs on transcription factors and function, we went back to the clone-based system and used Tfh clones derived from HD for subsequent analyses to elucidate the IFN influence on an isolated Tfh cell population. After 3 days of type I IFN exposure, αCD3/αCD28-activated Tfh clones showed the tendency of elevated expression of the Tfh-associated transcription factors Bcl6 and cMaf but also increased TCF-1 expression. Concurrently, type I IFNs increased the expression of the Th1 master regulator Tbet (**Figure 4D** bar graphs and heatmap). An induction of a Th1 bias was further reflected by the cytokine production. Indeed, αCD3/αCD28-stimulated Tfh clones showed increased IL-21 but also IFN-γ and TNF production after type I IFN exposure (**Figure 4E**). The presence of type I IFNs during coculture of Tfh clones with naïve autologous B cells revealed supportive effects on B cell helper capacity reflected by a tendency of elevated plasmablast formation (**Supplementary Figure 7G**) and increased concentrations of IgM and IgG in coculture supernatants (**Figure 4F**). The IgG levels in the supernatants correlated with the plasma cell frequency and the IL-21 production of the Tfh clones throughout the different IFN conditions (**Supplementary Figures 7H, I**) confirming the direct dependence of plasma cell differentiation and class switch on the individual IL-21 production in response to the respective IFN. Although the observed changes introduced by type I IFN stimulation were only of limited extend, **Figure 4G** clearly illustrates the unique profile of Tfh cells introduced by short-term type I IFN exposure including elevated activation, an induced Th1 bias characterized by elevated Tbet expression and IFN-γ production, but concurrently advanced Tfh features with higher IL-21 production and B cell helper capacity. These findings suggest that the characteristics of HCV clones could be correlated with chronic type I IFN exposure, although type I IFN stimulation did not result in a precise copy of the phenotype of HCV-specific CD4 T cells and the accompanying clones, particularly regarding CXCR3 and TCF-1 expression.

### Direct Signaling of Type I IFNs Contributes to Elevated cTfh and cTfh1 Cell Frequencies During Pegylated IFN-α Therapy of cHCV Infection

To elucidate the basis of the stronger effects elicited by type I in comparison to type II and III IFNs on the Tfh profile, we analyzed the direct signaling of the IFN types *via* STAT1 phosphorylation. Indeed, type I but not type II or III IFNs were able to directly signal to Tfh clones (**Figure 4H**). This finding also applied to the pegylated form of IFN-α (peg-IFN-α, Pegasys^®^), although the signaling capacity was decreased compared to unpegylated type I IFNs as described previously (49). In order to confirm our *in vitro* findings *in vivo*, we analyzed whether the therapeutic elevation of the systemic IFN-α concentration affected circulating Tfh population in historic samples from patients with cHCV undergoing peg-IFN-α therapy. Here, we found the frequency of cTfh as well as cTfh1cells to be significantly elevated, early after the initiation of peg-IFN-α therapy, while non-Tfh cells were unaffected (**Figure 4I**). However, the effect was temporary as the frequencies of both populations reached baseline levels around weeks 17 to 45 of therapy.

In summary, our results show stable Th1-biased Tfh-like characteristics (PD-1↑, CXCR3↑, Bcl6↑, Tbet↑, IL-21↑, IFN-γ↑) and superior Tfh functionality of HCV clones that were generated after elimination of chronic infection. Furthermore, we were able to show that type I IFNs elicit direct effects on fully differentiated Tfh cells including the enhancement of Tfh activation, phenotype and functionality (CD38↑, CXCR3↓, IL-21↑, B cell help↑) despite the concurrent induction of a Th1 bias (Tbet↑, IFN-γ↑). These short-term effects of type I IFNs on Tfh cells resemble the maintained characteristics of HCV clones. Based on these results, we suggest that chronic type I IFN exposure during cHCV infection *in vivo* played a key role in the induction and stabilization of the Th1 bias and the superior Tfh characteristics of HCV clones.

## Discussion

The development of a prophylactic vaccination remains an important aim for HCV elimination, since DAA-cured patients are not protected from HCV re-infection and the incidence remains high in individuals at risk (50–52). However, vaccination of DAA-cured patients faces additional challenges, since it targets HCV-specific T or B cells that have been exposed to their cognate antigen for years or decades during chronic infection and therefore have altered or diminished functionality (44, 53–55). For the most common vaccination strategies, which rely on the induction of a neutralizing antibody response, highly functional HCV-specific Tfh cells would be important mediators, as they support B cell differentiation and affinity maturation during the GC reaction (50). As any other CD4 T cell lineage, Tfh cells are a rather heterogenous T cell population. One of the most prominent subsets of cTfh cells are those with overlapping Th1 characteristics, often referred to as cTfh1 cells. They have been shown to correlate with antibody responses during infections with HCV and HIV (14–18, 24) and after vaccination (20–23) and are the dominant subset within circulating HCV-specific CD4 T cells after DAA therapy (26). However, studies of their actual B cell helper capacity were hampered by the scarcity of this cell population and consequently limited to the bulk level. These analyses have revealed controversial results, showing either superior B cell help for CXCR3- Tfh cells (12, 13) or an equal level of B cell support in CXCR3- Tfh cells and CXCR3+ Tfh1 cells (16, 25).

Using a clone-based system, we were able to overcome methodological restrictions associated with the low cell counts to directly analyze the functionality of HCV-specific CD4 T cells *in vitro* (HCV clones), demonstrating superior B cell helper capacity despite a Th1-bias in the phenotype with concurrent PD-1 and CXCR3 surface expression and high levels of Bcl6 and Tbet, as well as the cytokine production with high IL-21 and IFN-γ production. Influenza-specific CD4 T cell clones (Flu clones) and Tfh and Th1 clones with unknown antigen-specificity were used as a reference system. We ensured direct comparability of the clone types and analyses by synchronized cell culture conditions and treatment as well as the parallel analysis of all CD4 T cell clones in one experimental run. IL-2 stimulation during cell culture was reduced to low levels to balance proliferation and the inhibitory effects of IL-2 signaling on the Tfh program (9, 34, 56). Furthermore, we could exclude an individual induction of Treg features by IL-2 (43), since the clone types showed equal FoxP3 expression levels. Eventually, stabile lineage-specific differences *in vitro* including higher PD-1 and IL-21 and lower CXCR3 and IFN-γ as well as higher B helper capacity of Tfh compared to Th1 clones validated the clone-based approach.

The expression of TCF-1 in the clone types was relevant in two contexts, since it is a key regulator during Tfh differentiation upstream of Bcl6 (38, 39) and also drives the maintenance of CD8 T cell effector functions during chronic viral infections (40–42, 57). HCV and Flu clones showed reduced TCF-1 expression in comparison to Tfh and Th1 clones. Also the higher expression of TCF-1 in Th1 clones was unexpected but could likely be an effect of the IL-2-rich culture conditions, since IL-2 is known to elicit inhibitory effects on the Tfh program and TCF-1 expression (58, 59) and Tfh cells are especially sensitive to IL-2 influence (34). In summary, TCF-1 expression analysis led to inconclusive results in our study.

After peptide stimulation, HCV and Flu clones showed highly variable cytokine production with only 40-60% of the population producing IL-21 or IFN-γ. A clone, although derived from a single cell, is still a heterogenous cell population within the culture e.g. in context of vitality, cell cycle, metabolic state or activation affecting the expression of surface markers like the T cell receptor (TCR) or HLA-DR (**Supplementary Figure 4**) and thus the percentage of cells in culture presenting and responding to peptide and also to αCD3/αCD28 stimulation. As control the TNF production was also determined, which was around 80% after peptide stimulation (**Supplementary Figure 6**), confirming the antigen-specificity of the clones.

Despite *in vitro* expansion, HCV-specific CD4 T cell clones still have been a very limited resource throughout our study. Due to technical limitations including diminished expansion capacity of antigen-specific CD4 T cells or a limited number of donors with matched HLA alleles, we included HCV clones from three DAA-treated patients and Flu clones from one healthy individual (**Table 1** and **Supplementary Table 1**). It is possible, that the limited number of individuals included in our study introduced a bias within our results. However, within one patient, we considered the individuality of the clones that derived from a single HCV-specific CD4 T cell within a heterogeneous tetramer-positive population as an additional level of control and thus aimed to include more than one clone per patient.

In comparison to HCV clones, Flu clones were not able to support naïve B cells during co-culture, confirming previous results of cTfh1 *ex vivo* analysis after Flu vaccination (20). Moreover, Flu clones were not able to maintain CXCR3 (and in 3 cases also PD-1, **Table 3**) expression *in vitro* indicating a lower level of stabilization within a certain phenotype.

The combined analysis of all analyzed parameters including surface marker and transcription factor expression, cytokine production and B cell helper capacity showed distinct characteristics of HCV and Flu clones, suggesting an influence of the immunologic environment *in vivo* on the characteristics that the respective CD4 T cell clones will maintain and display *in vitro*. We hypothesize, that the phenotype and functionality of HCV clones as well as the stability of these characteristics are the result of the immunologic environment of cHCV infection involving chronic exposure to antigen and IFN-stimulation. This would also be in line with observations in CD8 T cells, which show an irreversibly imprinted signature (44, 45, 60, 61) after chronic viral infection which is based on epigenetic reprogramming. A further demonstration of the stabilization of CD4 T cell features was the maintenance of basic Tfh and Th1 characteristics within Tfh and Th1 clones confirming previous findings of lineage commitment in mice during persistent LCMV infection (8). The stability of CD4 T cell characteristics in our model could also be based on epigenetic alterations (8, 44), however, we did not address this point, since the *ex vivo* epigenetic analysis provides more unbiased results and therefore would be more relevant in this context.

Collectively, despite the bias of using phenotypically altered *in vitro*-expanded cells, we could confirm our *ex vivo* results on HCV-specific CD4 T cells that showed an overlapping Tfh/Th1 phenotype and gene expression profile (26) and we were able to demonstrate superior Tfh functionality of these cell population on the antigen-specific level. Our results strongly support previous findings that the B cell or the neutralizing antibody response to HCV infection are positively correlated to the Tfh1 rather than Tfh response (16–18, 24).

The mechanisms that drive Tfh1 development remain elusive and flexibility between the phenotypes and a mixed phenotype as a transitional state during differentiation are discussed [reviewed in (62)]. The stability of HCV clone characteristics *in vitro* additionally suggests the Tfh/Th1 overlap as a fixed phenotype that is shaped during chronic viral infection in humans. Since functional Tfh cells accumulate in the IFN-rich milieu of HCV-infected liver (10, 11, 46), we investigated IFN exposure as possible mediator of Tfh maintenance and stabilization. It is well established that Tfh cells accumulate during persistent infections with poorly or non-cytopathic viruses that are characterized by a type I IFN response [reviewed in (1, 63)]. Thereby, type I IFNs affect CD4 T cell priming dependent on the stage of infection (64, 65). In the persistent phase Th1 differentiation is suppressed by dendritic cells in response to type I IFN signals and CD4 T cell almost exclusively differentiate into Tfh cells (64, 66). Moreover, type I IFNs also directly induced Tfh features in murine naïve CD4 T cells *in vitro* including Bcl6, CXCR5 and PD-1 expression (67). In contrast, in human *in vitro* experiments type I IFNs failed to induce CXCR5 expression in naive CD4 T cells as a surrogate of Tfh differentiation (68). However, direct effects of type I IFN signaling on the maintenance of differentiated Tfh cells have not been described yet.

In our analyses type I IFNs enhanced Tfh features but also induced a Th1 bias, which was mediated by direct signaling of type I IFNs to Tfh cells *via* STAT1 but the changes were not of great extend and most of the analyzed parameters were significantly up-regulated when compared to type 2 and 3 IFN stimulation but not when compared against the control condition without IFN stimulation. Since we had to counterbalance IFN-induced cell death, we kept the exposure times to the IFNs as short as possible, which might have been insufficient to introduce more significant changes when compared to a decade-long exposure during chronic viral infection.

Moreover, the upregulation of CD127 on Tfh cells after type I IFN stimulation seemed contradictory to the downregulation of CXCR3, since CD127 is not expressed on activated effector GC Tfh cells (69) but CXCR3- cTfh cells are known to share features of GC Tfh cells (12). We think that by using PBMCs for our experiment, the results cannot be related to the complex environment of the GC. Moreover, PBMCs contain further immune cell populations, which are absent in the GC, but can co-stimulate the Tfh cells in response to IFN stimulation *in vitro*. However, since IL-7 signaling is involved in Tfh development and regulation (70–72) and type I IFN influence can inhibit IL-7 signaling in CD4 T cells (73) upregulation of the IL-7 receptor α (CD127) could be a compensatory response during type I IFN stimulation.

In summary, our results suggest a correlation of type I IFN exposure and the emergence of a Tfh1 phenotype. Moreover, we could not only confirm the elevation of peripheral bulk Tfh cells (CXCR5+) during peg-IFN-α therapy (74) using a more precise definition including CXCR5 and PD-1 but also observed an elevation of the cTfh1 population, supporting a positive correlation of the systemic type I IFN concentration and peripheral Tfh and Tfh1 cells *in vivo*. Further studies are necessary to clarify the role of type I IFNs for Tfh maintenance and phenotype during chronic viral infection.

For our coculture setup, we used naïve B cells, although it is unlikely, that Tfh cells interact with naïve B cells in the GC. This was a methodological decision, since naïve B cell activation and differentiation require more time and more complex signal combinations and thus were a better measure for the B cell helper capacity of a Tfh clones. As control we tested the coculture with memory B cells and observed similar tendencies of regulations after IFN stimulation compared to coculture with naïve B cells (**Supplementary Figure 8**).

It has been reported that IFN-α inhibits IL-2-induced proliferation of CD4 T cells (73) by downregulation of CD25, the high affinity IL-2 receptor (75) and thus is able to interfere with IL-2 signaling what could counteract the negative effects of the IL-2 on the Tfh program (9, 56, 58, 59, 76, 77) and thus stabilize or even enhance the Tfh characteristics. On the other hand, IFN-α increased the IFN-γ production in CD4 T cells (78) reviewed in (79) further supporting our observations of a concurrent induction of Th1-like features in Tfh clones. Based on our results, we suggest that type I IFNs are involved in the maintenance, stabilization and even enhancement of Tfh features despite the induction of a Th1 bias although *in vitro* type I IFN stimulation of Tfh clones did not precisely reproduce the phenotype of HCV clones. The reduced CXCR3 expression as well as the elevated TCF-1 expression after 3 d type I IFN exposure were contradictory to our observations in HCV clones, which showed high CXCR3 and low TCF-1 expression. These differences could also be a result of the shorter exposure time to the IFNs or the limited complexity of the single IFN stimulation *in vitro* which is not comparable to an IFN response *in vivo*. However, these regulations also further supported the enhancement of Tfh features by type I IFNs as cTfh cells that most closely resemble GC Tfh cells are CXCR3- (12) and TCF-1 is involved in regulation of Bcl6 expression (38).

Although *in vitro* analysis can only provide limited insight into the *in vivo* situation, the clone-based approach offered the unique opportunity to evaluate the functionality of HCV-specific CD4 T cells and provided the experimental tools to investigate the IFN influence on an isolated Tfh population.

Collectively, we were able to demonstrate superior B cell helper capacity as a stabilized feature of HCV-specific Tfh1 cells. Furthermore, our results suggest that chronic exposure to type I IFN signaling could be involved in Tfh stabilization and induction of Th1-skewed characteristics during chronic HCV infection. The high Tfh functionality of HCV-specific CD4 T cells may be targeted by future vaccination design aiming to introduce a neutralizing antibody response.

## Data Availability Statement

Raw data is available upon reasonable request. Further supporting data are available from the corresponding authors upon reasonable request. All requests for raw and analyzed data and materials will be reviewed by the corresponding authors to verify if the request is subject to any intellectual property or confidentiality obligations. Any data and materials that can be shared will be released via a Material Transfer Agreement. R code and according data tables are available online: https://github.com/teximmed2-fr/HCV-clones.

## Ethics Statement

The studies involving human participants were reviewed and approved by ethics committee of the Albert Ludwig University Freiburg (344/13, 507/19 and 227/15) Engelberger Straße 21 79106 Freiburg E-Mail: ekfr@uniklinik-freiburg.de. The patients/participants provided their written informed consent to participate in this study.

## Author Contributions

Study concept and design, KZ and TB. Acquisition of data, KZ, SK, SE, KW, MS, and MR. Analysis and interpretation of data, KZ, SK, SE, and TB. Drafting of the manuscript, KZ, SE, and TB. Critical revision of the manuscript for important intellectual content, TB, RT, and MH. Statistical analysis, KZ, SK, SE, and AG. Obtained funding, TB, MH, and RT. Study supervision, RT and TB. All authors contributed to the article and approved the submitted version.

## Funding

This work was supported by grants from the Deutsche Forschungsgemeinschaft (DFG, German Research Foundation) – project 272983813 – TP01 to RT, TP04 to TB, TP20 to MH. The project was also supported by the DZIF (German Center for Infection Research).

## Conflict of Interest

The authors declare that the research was conducted in the absence of any commercial or financial relationships that could be construed as a potential conflict of interest.

## Publisher’s Note

All claims expressed in this article are solely those of the authors and do not necessarily represent those of their affiliated organizations, or those of the publisher, the editors and the reviewers. Any product that may be evaluated in this article, or claim that may be made by its manufacturer, is not guaranteed or endorsed by the publisher.
