# Supplementary material for: Th1-Biased Hepatitis C Virus-Specific Follicular T Helper-Like Cells Effectively Support B Cells After Antiviral Therapy
Source: Front Immunol. 2021 Sep 30;12:742061. doi: 10.3389/fimmu.2021.742061 (PMC8514946; doi:10.3389/fimmu.2021.742061)
Supplement: Supplementary file 1 [file DataSheet_1.docx]

**Supplementary Materials and Methods**

**Isolation of PBMCs**

Peripheral blood mononuclear cells (PBMCs) were isolated from EDTA anti-coagulated blood with density gradient centrifugation using human lymphocyte separation medium (anprotec, Germany) according to the manufacturer’s instructions.

**Preparation of feeder cells**

Whole blood samples were obtained from healthy volunteers and PBMCs were isolated as described above. After counting the desired amount of PBMCs were transferred into a new centrifuge tube and exposed to 30 Gy γ-irradiation. Feeder cells were washed and resuspended in the desired amount of cell culture medium.

**Generation of CD4 T cell clones**

Whole blood samples were obtained from cHCV patients or HD and PBMCs were isolated as described above. The phenotypes of the cloned cells from all clone types are summarized in Supplementary Tables 2 and 3. Tfh and Th1 clones with unknown antigen-specificity from cHCV patients or HD were generated *ex vivo* from isolated PBMCs after surface staining and single cell sorting. HCV-specific CD4 T cell clones (HCV clones) were generated after antigen-specific expansion of 2x10^6^ PBMCs with 5 µM HCV-specific peptide (Supplementary Table 3) and an αCD28 antibody for costimulation in 1 ml RPMI supplemented with 10 % human serum (AB male, PAN-Biotech, Germany), 2 mM Glutamin (ThermoFisher Scientific Inc., Germany), 100 U/ml penicillin and 100 µg/ml streptomycin. The cultures were incubated at 5 % CO_2_ and 37 °C for 14 d. 10 U/ml IL-2 were added on days 3, 7 and 10. Half of the medium was changed on demand and IL-2 was added accordingly. On day 14 the cells were stained with surface antibodies and tetramers as described below and CD4+, Tet+ single cells were sorted on a FACSMelody (BD Biosciences, Germany). Due to cloning after peptide-specific expansion nodefined information on the ex vivo-phenotype of HCV clones is available. Influenza-specific CD4 T cell clones (Flu clones) were generated from HD PBMCs after magnetic bead-based enrichment of antigen-specific CD4 T cells and surface stain as described previously (1). CD45RA- CD4+, Tet+ cells were single cell index sorted on a FACSARIA Fusion (BD Biosciences). Single cells were sorted into one well of a 96-well plate filled with 100 µl medium and 200.000 feeder cells. After sorting PHA and IL-2 were added in medium to final concentrations of 40 µg/ml and 20 U/ml, respectively. All wells were supplemented with medium and IL-2 every 3 to 4 days. On day 14 after cloning all wells were transferred to 48-well plates and kept in the same conditions until visible cell proliferation and subsequent further transfer. After the clones were established, the culture conditions were adapted to serum-free conditions using AIM-V cell culture medium (ThermoFisher Scientific Inc.) and the concentration of IL-2 was reduced to 5 U/ml. Antigen-specificity of the virus-specific CD4 T cell clones was verified by tetramer staining. Representative stainings are shown in Supplementary Fig. 4.

**Flow cytometry**

Used peptide and tetramers as well as antibodies are summarized in Supplementary Tables 3 and 4, respectively. Between 1x10^5^ and 5x10^5^ cells were stained with tetramers and antibodies in 96-well V-bottom plates. Cells were incubated with 0.5 µl of the according tetramers for 30 min at 4 °C. After two washing steps in staining buffer (PBS + 1 % fetal calf serum (FCS)) surface antibody mixtures were added and incubated for 15 min at room temperature (RT). Fixable Viability dye^eFluor780^ (eBioscience, Germany) was used to exclude dead cells. Cells were then washed again and fixed in fixation buffer (PBS + 4 % (w/v) paraformaldehyde).

For intracellular staining of transcription factors cell were stained with surface antibodies as described above. The cells were then fixed, permeabilized and stained with intracellular antibodies using the FoxP3 staining buffer set (ThermoFisher Scientific Inc.) according to the manufacturer’s instructions. Cells were then washed in the according buffer and resuspended in fixation buffer.

Prior to intracellular staining of cytokines 5x10^5^ cells were stimulated with 5 µM their respective peptide or αCD3 / αCD28 (T cell activator) for 5 h or PMA/Ionomycin for 3.5 h in cell culture medium in presence of 0,325 µl/ml GolgiStop^TM^ and 0,5 µl/ml GolgiPlug^TM^ (BD Biosciences). For peptide stimulation no additional antigen-presenting cells were added, since the CD4 T cell clones expressed HLA-DR (Supplementary Fig. 4C), due to their highly activated state during *in vitro* cultivation (2) and thus were able to self-present the peptide. The cells were then washed, fixed, permeabilized and stained with intracellular antibodies using Cytofix/Cytoperm^TM^ solutions (BD Biosciences) according to the manufacturer’s instructions. Cells were then washed in the according buffer and resuspended in fixation buffer. Unstimulated cells served as control. Background production was not subtracted, since the highly activated state of the clone cells, caused by the culture conditions, already resulted in elevated background production in some cases. We therefore focused on the overall (not the specific) levels of cytokine production after stimulation.

For the staining of phosphorylated STAT1 1 x 10^6^ clone cells for each condition were seeded per well in deep-well plates and incubated for 2 h at 5 % CO_2_ and 37 °C. Stimulation was performed for 20 min with the different IFNs in presence of the surface antibodies. Subsequently, phosphorylated STAT1 was stained with a BD Phosflow™ antibody according to the manufacturer’s instructions.

Stained cells were measured with an LSR Fortessa (BD Biosciences). FlowJo 10.0.7 (LLC, BD Life Sciences, USA) was applied for the analysis of flow cytometric data. Gating strategies are shown in Supplementary Fig. 1 and 2.

**Antiviral activity of the used IFN reagents**

For analysis of the antiviral activity stably transfected Huh7 cells (Luc A2 JFH) were used. The replicon contained the genotype *2a* (*JFH*-1 strain) genome, a neomycin resistance for selection and luciferase as reporter (3). Cells were cultivated in DMEM supplemented with 10 % FCS, 100 U/ml penicillin, 100 µg/ml streptomycin and non-essential amino acids and kept in a semi-confluent state at 5 % CO_2_ and 37 °C. For selection of cells bearing the replicon, 0.5 mg/ml G418 (ThermoFisher Scientific Inc.) were added to the cell culture.

For the assay the cells were detached from cell culture surface using EDTA solution. The cells were washed twice with medium. 30,000 cells per well were seeded in a 24-well plate in 1 ml medium (see above). After overnight incubation, 0.5 pmol/ml of the different IFNs were added and the cells were incubated for 3 days. SteadyGlo® reagent (Promega Corporation, USA) was used to determine luciferase activity according to the manufacturer’s instructions. The lysate was transferred in white 96-well plates and subsequently luminescence was measured with a TECAN Spark Multiwell Plate Reader (TECAN GmbH). For each condition duplicates were analyzed. The condition without IFN served as control.

**References**

1. Smits M, Zoldan K, Ishaque N, Gu Z, Jechow K, Wieland D, et al. Follicular T helper cells shape the HCV-specific CD4+ T cell repertoire after virus elimination. *J Clin Invest* (2020) **130**:998–1009. doi:10.1172/JCI129642

2. Holling TM, van der Stoep N, Quinten E, van den Elsen, Peter J. Activated human T cells accomplish MHC class II expression through T cell-specific occupation of class II transactivator promoter III. *J Immunol* (2002) **168**:763–70. doi:10.4049/jimmunol.168.2.763

3. Lohmann V, Körner F, Koch J, Herian U, Theilmann L, Bartenschlager R. Replication of subgenomic hepatitis C virus RNAs in a hepatoma cell line. *Science* (1999) **285**:110–3. doi:10.1126/science.285.5424.110

**Supplementary Figures**

**Supplementary Figure 1: Gating strategy of CD4+ clone cells.** Representative staining and gating strategy for determination of surface marker and transcription factor expression as well as cytokine production within CD4+ clone cells (Fig. 1-3). FSC: forward scatter, SSC: sideward scatter, Via: fixable viability dye

**Supplementary Figure 2: Gating strategy of PBMCs after 3 d IFN exposure.** Representative staining and gating strategy for characterization of non-Tfh and Tfh cells (red gates) within isolated HD PBMCs after 3d incubation in the presence of IFNs (Fig. 4 A-B). The same gates for activation and costimulatory marker expression were applied to non-Tfh cells. Via: fixable viability dye

**Supplementary Figure 3: Antiviral activity of the used interferon reagents.** For quality control the antiviral activity of 0.5 pmol/ml of the interferons was analyzed using Huh7 cells containing an HCV genotype 2a replicon with luciferase as reporter. Antiviral activity was measured by reduction of luciferase activity. RLU: relative light units

**Supplementary Figure 4: Antigen-specificity and HLA-DR expression of HCV and Flu clones.** Representative pseudocolor plots of tetramer-stainings of (A) an HCV clone and (B) a Flu clone with the respective histograms on the left. (C) HLA-DR expression of HCV- (n = 18) and Flu-specific CD4 T cell clones (n = 5) with representative histogram and pseudocolor plot of a stained HCV clone on the right.

**Supplementary Figure 5: Parameters that distinguish Tfh and Th1 clones.** (A) Surface markers shown in Fig. 1 (n = 12 Tfh and 15 Th1 clones). (B) Cytokine production shown in Fig. 2 (n = 15 Tfh and 13 Th1 clones). (C) B cell helper capacity shown in Fig. 2 (n = 14 Tfh and 11 Th1 clones). Mann-Whitney rank sum test was used to compare two groups. *p < 0.05; **p < 0.01

**Supplementary Figure 6: Cytokine production of CD4 T cell clone types.** Cytokine production of the CD4 T cell clone types was determined by flow cytometry after stimulation with their respective peptide (n = 17 HCV, 15 unstim. HCV, 5 Flu, 5 unstim. Flu clones), αCD3 / αCD28 (n = 15 HCV, 5 Flu, 15 Tfh, 13 Th1) or PMA / Iono (n = 17 HCV, 5 Flu, 15 Tfh, 13 Th1 clones). Representative pseudocolor plots are shown on the right side of the bar graphs. Kruskal-Wallis test with Dunn’s test for multiple comparisons was applied to compare differences between all groups to HCV clones as control. Mann-Whitney rank sum test was used to compare two groups.

**Supplementary Figure 7: T cell-B cell-coculture to evaluate B helper capacity *in vitro*.** Isolated naive B cells were incubated with αCD3 / αCD28-activated CD4 T cell clones. After 7 d immunoglobulin levels in culture supernatants were determined by ELISA. (A) Validity of the system was tested by correlation of Ig levels in coculture supernatants and plasmablast frequency after 7 d of autologous coculture (n = 10). (B) Representative staining of a Tfh clone and differentiated B cells after 7 d of autologous coculture from the same experimental run in (A). Transitional B cells are shown additionally to illustrate the dynamics of the B cell development in this *in vitro* system. (C) Positive and negative controls for B cell functionality. Naïve B cells of one HD were incubated for 7 d in the indicated conditions. Symbols represent one replicate. The same naïve B cells were used for all allogeneic cocultures in Fig. 3. (D) To exclude influences by the allogeneic setup of the coculture Flu-specific CD4 T cell clones were incubated with autologous naïve B cells as in-run control to the experiment in Fig. 2. One symbol represents the mean of duplicates. Comparable results were achieved with both coculture setups. Negative (E) and positive controls (F) for B cell functionality after IFN exposure. Naïve B cells of 3 HD were incubated for 7 d in the indicated conditions. Symbols represent the mean of duplicates. Bars represent the median. The same naïve B cell preparations were used for the cocultures in Fig. 4. (G) Plasmablast frequencies (bars represent medians) after coculture in presence of IFNs (n = 2). Representative stainings on the left. (H) Spearman correlation of plasmablast frequency (n = 2 clones) and (I) IL-21 production of Tfh clones (n = 6 clones) with Ig concentrations in the coculture supernatants throughout the different IFN conditions.

**Supplementary Figure 8: T cell-B cell-coculture with memory B cells to evaluate B helper capacity *in vitro*.** Sorted autologous memory B cells were incubated with αCD3 / αCD28-activated Tfh clones (n = 2) of a healthy donor (HD). After 7 d immunoglobulin levels in culture supernatants were determined by ELISA. (A) Negative controls for B cell functionality. B cells of the HD were incubated for 7 d in the indicated conditions. Symbols represent one replicate. (B) Co-culture of memory B cells and Tfh clones in the indicated conditions. Symbols represent the mean of duplicates. (A, B) Bars represent the median. ROF: roferon

**Supplementary Figure 9: Correlation matrix of all parameters analyzed in Figure 3.** Spearman correlation was used to analyze correlations among phenotypic and functional parameters. (A) Correlation matrix of all analyzed parameters. (B) Correlation matrix of parameters that are able to discriminate between Tfh and Th1 clones (Supplementary Fig. 5). (A, B) The heatmap coloring represents the Spearman R, #: p ≤ 0.05). Only clones with a complete analysis of all parameters were included (n = 15 HCV, 5 Flu, 11 Tfh, 10 Th1 clones).

**Supplementary Tables**

**Supplementary Table 1: CD4 T cell clones**

| **clone number** | **clone:** E: generated after *in vitro* peptide-specific expansion, donor number, antigen, original target well | **Surface markers** | **transcription factor expression** | **cytokine production** | | | **B cell helper capacity** | **transcription factor expression after IFN incubation** | **cytokine production after IFN incubation** | **pSTAT** | **B cell helper capacity after IFN incubation** |
| --- | --- | --- | --- | --- | --- | --- | --- | --- | --- | --- | --- |
|  |  |  |  | **CD3 / CD28** | **PMA / Iono** | **peptide** |  |  |  |  |  |
| 1 | E14HCVB4 | x | x | x | x | x | x |  |  |  |  |
| 2 | E16HCVIA4 | x | x | x | x | x | x |  |  |  |  |
| 3 | E16HCVIE12 | x | x |  |  |  | x |  |  |  |  |
| 4 | E16HCVIG1 | x | x | x | x | x | x |  |  |  |  |
| 5 | E16HCVIIG11 | x | x | x | x | x | x |  |  |  |  |
| 6 | 18HCVA9 | x | x | x | x | x | x |  |  |  |  |
| 7 | 18HCVB5 | x | x | x | x | x | x |  |  |  |  |
| 8 | E18HCVA12 | x | x | x | x | x | x |  |  |  |  |
| 9 | E18HCVA6 | x | x | x | x | x | x |  |  |  |  |
| 10 | E18HCVA8 | x | x | x | x | x | x |  |  |  |  |
| 11 | E18HCVB10 | x | x | x | x | x | x |  |  |  |  |
| 12 | E18HCVB3 | x | x | x | x | x | x |  |  |  |  |
| 13 | E18HCVB7 | x | x | x | x | x | x |  |  |  |  |
| 14 | E18HCVC2 | x | x | x | x | x | x |  |  |  |  |
| 15 | E18HCVC7 | x | x |  | x | x |  |  |  |  |  |
| 16 | E18HCVD4 | x | x |  | x | x |  |  |  |  |  |
| 17 | E18HCVD5 | x | x | x | x | x | x |  |  |  |  |
| 18 | E18HCVE12 | x | x | x | x | x | x |  |  |  |  |
| 19 | 2FluA7 | x | x | x | x | x | x |  |  |  |  |
| 20 | 2FluA9 | x | x | x | x | x | x |  |  |  |  |
| 21 | 2FluC1 | x | x | x | x | x | x |  |  |  |  |
| 22 | 2FluE2 | x | x | x | x | x | x |  |  |  |  |
| 23 | 2FluH2 | x | x | x | x | x | x |  |  |  |  |
| 24 | 2TfhA2 | x | x | x | x |  | x | x |  | x | x |
| 25 | 2TfhA9 | x | x | x | x |  | x | x | x |  | x |
| 26 | 2TfhB2 |  |  |  |  |  |  | x |  |  | x |
| 27 | 2TfhC7 |  |  |  |  |  |  | x |  |  | x |
| 28 | 2TfhD11 |  |  |  |  |  |  |  | x |  | x |
| 29 | 2TfhD3 |  |  |  |  |  |  |  | x |  |  |
| 30 | 4TfhIIF5 |  |  |  |  |  |  |  | x |  |  |
| 31 | 4TfhVIE3 | x | x | x | x |  | x |  | x |  |  |
| 32 | 3TfhA11 |  |  |  |  |  |  |  | x |  | x |
| 33 | 3TfhA5 |  |  |  |  |  |  | x | x |  | x |
| 34 | 3TfhB1 |  |  |  |  |  |  | x |  |  | x |
| 35 | 3TfhB6 | x | x | x | x |  | x |  |  |  | x |
| 36 | 3TfhC4 | x | x | x | x |  | x | x | x | x | x |
| 37 | 3TfhD10 |  |  |  |  |  |  | x |  |  | x |
| 38 | 2Th1E10 | x | x | x | x |  | x |  |  |  | x |
| 39 | 4Th1IIH3 | x | x | x | x |  | x |  |  |  |  |
| 40 | 4Th1VIH6 | x | x | x | x |  | x |  |  |  |  |
| 41 | 3Th1E11 | x | x | x | x |  | x |  |  |  |  |
| 42 | 3Th1E12 | x | x | x | x |  | x |  |  |  |  |
| 43 | 15TfhB3 | x | x | x | x |  | x |  |  |  |  |
| 44 | 15TfhG4 | x | x | x | x |  | x |  |  |  |  |
| 45 | 15TfhG5 | x | x | x | x |  | x |  |  |  |  |
| 46 | 16TfhA3 |  | x | x | x |  | x |  |  |  |  |
| 47 | 16TfhC6 |  |  | x | x |  | x |  |  |  |  |
| 48 | 16TfhD3 | x | x | x | x |  | x |  |  |  |  |
| 49 | 17TfhB1 | x | x | x | x |  | x |  |  |  |  |
| 50 | 17TfhC7 | x | x | x | x |  |  |  |  |  |  |
| 51 | 18TfhB12 |  | x | x | x |  | x |  |  |  |  |
| 52 | 18TfhB3 | x | x | x | x |  | x |  |  |  |  |
| 53 | 15Th1B9 | x |  | x | x |  | x |  |  |  |  |
| 54 | 15Th1D9 | x | x | x | x |  |  |  |  |  |  |
| 55 | 16Th1E1 | x | x | x | x |  | x |  |  |  |  |
| 56 | 16Th1E5 | x | x |  |  |  |  |  |  |  |  |
| 57 | 16Th1F1 | x |  |  |  |  |  |  |  |  |  |
| 58 | 17TH1F4 | x | x | x | x |  |  |  |  |  |  |
| 59 | 17TH1H6 | x | x | x | x |  | x |  |  |  |  |
| 60 | 18Th1E8 | x | x | x | x |  | x |  |  |  |  |
| 61 | 18Th1G5 | x | x | x | x |  | x |  |  |  |  |
| 62 | 18Th1G9 | x | x | x | x |  | x |  |  |  |  |
| 63 | 1TfhIC1 |  |  |  |  |  |  |  |  |  | x |
| 64 | 1TfhIE1 |  |  |  |  |  |  |  |  |  | x |

**Supplementary Table 2: Cytokines and stimulants**

| **Reagent** | **Provider** | **relevant additional information** |
| --- | --- | --- |
| recombinant human Interleukin 2 | Stemcell Technologies | 5x10^5^ U/mg |
| recombinant human Interferon IFN-alpha 2A | Stemcell Technologies | 19.4 kDa |
| recombinant human Interferon IFN-beta | Stemcell Technologies | 20.0 kDa |
| recombinant human Interferon IFN-gamma | Stemcell Technologies | 17.0 kDa |
| recombinant human Interferon IFN-lambda 1 | BioLegend | 19.8 kDa |
| recombinant human Interferon IFN-lambda 2 | BioLegend | 20.8 kDa |
| recombinant human Interferon IFN-lambda 3 | R&D Systems | 20.1 kDa |
| recombinant human Interferon IFN-omega | eBioscience | 20.0 kDa |
| Phytohemagglutinin (PHA) | Sigma-Aldrich | mucoprotein form |
| human T cell activator | Stemcell Technologies | - |
| αCD28 antibody | BD Biosciences | - |
| recombinant human MEGACD40L ® | Enzo Life Sciences | - |
| recombinant human Interleukin 21 (carrier-free) | BioLegend | - |
| Pegasys ® | Roche Pharma AG | 60 kDa, 180 µg/ 0.5 ml |
| Roferon | Roche Pharma AG | - |

**Supplementary Table 3: peptide sequences and according tetramers.** Phycoerythrin (PE)-labeled major histocompatibility complex (MHC) class II-tetramers were obtained from MBL, Woburn, United States.

| **peptide sequence** | **HLA molecule** | **Virus** | **Protein** |
| --- | --- | --- | --- |
| TLLFNILGGWVAA | DRB1*01:01 | HCV | NS4b |
| GINAVAYYRGLDVSV | DRB1*15:01 | HCV | NS3 |
| PKYVKQNTLKLAT | DRB1*01:01 | Flu | HA |

**Supplementary Table 4: antibodies for flow cytometry**

| **Antigen** | **Fluorophore** | **Clone** | **Provider** | **Staining** |
| --- | --- | --- | --- | --- |
| Bcl6 | PE | K112-91 | BD Biosciences | intracellular |
| BTLA | APC | [MIH26](https://www.biolegend.com/en-gb/search-results?Clone=MIH26) | BioLegend | extracellular |
| CCR7 | PE-Dazzle | G043H7 | BioLegend | extracellular |
| CD134 (OX40) | PE-Cy7 | ACT-35 | BD Biosciences | extracellular |
| CD127 | BV605 | A019D5 | BioLegend | extracellular |
| CD14 | APC-eFluor780 | 61D3 | eBioscience | extracellular |
| CD19 | APC-eFluor780 | HIB19 | eBioscience | extracellular |
| CD19 | V500 | HIB19 | BD Biosciences | extracellular |
| CD20 | PE-Cy7 | 2H7 | eBioscience | extracellular |
| CD25 | BB515 | 2A3 | BD Biosciences | extracellular |
| CD27 | PE | O323 | eBioscience | extracellular |
| CD38 | BUV737 | HB7 | BD Biosciences | extracellular |
| CD4 | BV786 | L200 | BD Biosciences | extracellular |
| CD4 | BV510 | SK3 | BD Biosciences | extracellular |
| CD4 | FITC | RPA-T4 | BD Biosciences | extracellular |
| CD45RA | PerCP Cy5.5 | HI100 | eBioscience | extracellular |
| CD45RO | FITC | UCHL1 | eBioscience | extracellular |
| CD8 | APC-eFluor780 | SK1 | eBioscience | extracellular |
| cMaf | PerCPeFluor710 | symOF1 | Invitrogen | intracellular |
| CXCR3 | APC | 1C6/CXCR3 | BD Biosciences | extracellular |
| CXCR3 | BV510 | G025H7 | Biolegend | extracellular |
| CXCR3 | BUV395 | 1C6/CXCR3 | BD Biosciences | extracellular |
| CXCR5 | BV421 | J252D4 | BioLegend | extracellular |
| CXCR5 | APC | J252D4 | BioLegend | extracellular |
| FOXP3 | APC | PCH101 | eBioscience | intracellular |
| ICOS | BV711 | DX29 | BD Biosciences | extracellular |
| ICOS | PE-Cy7 | ISA-3 | eBioscience | extracellular |
| IFN-γ | BV510 | 4S.B3 | BioLegend | intracellular |
| IFN-γ | V450 | B27 | BD Biosciences | intracellular |
| IL-17A | BV605 | BL168 | BioLegend | intracellular |
| IL-2 | PE | MQ1-17H12 | BD Biosciences | intracellular |
| IL-2 | PE-eFluor610 | MQ1-17H12 | eBioscience | intracellular |
| IL-21 | Ax647 | 3A3-N2.1 | BD Biosciences | intracellular |
| IL-21 | PE | 3A3-N2.1 | BD Biosciences | intracellular |
| IL-4 | PE-Cy7 | 8D4-8 | BD Biosciences | intracellular |
| IL-6 | Ax700 | MQ2-13A5 | eBioscience | intracellular |
| OX40 | PE | Ber-ACT35 | BioLegend | extracellular |
| PD1 | PE | eBioJ105 | eBioscience | extracellular |
| PD-1 | BB515 | EH12.1 | BD Biosciences | extracellular |
| PD-1 | BV786 | EH12.1 | BD Biosciences | extracellular |
| PD-1 | BV421 | EH12.2H7 | BioLegend | extracellular |
| STAT1 | PE | 4a | BD Biosciences | intracellular |
| Tbet | BV421 | O4-46 | BD Biosciences | intracellular |
| Tbet | Pe-Cy7 | O4-46 | BD Biosciences | intracellular |
| TCF-1 | AlexaFluor488 | C63D9 | Cell signaling | intracellular |
| TIGIT | PE-Cy7 | MBSA43 | eBioscience | extracellular |
| Tim-3 | BV421 | F38-2E2 | BioLegend | extracellular |
| TNF | FITC | MAb11 | BD Biosciences | intracellular |
